# Knowledge, attitudes and practices regarding rabies risk in community members and healthcare professionals: Pétionville, Haiti, 2013

**DOI:** 10.1017/S0950268816003125

**Published:** 2017-03-14

**Authors:** N. FENELON, P. DELY, M. A. KATZ, N. D. SCHAAD, A. DISMER, D. MORAN, F. LARAQUE, R. M. WALLACE

**Affiliations:** 1 Department of Epidemiology and Laboratory Research, Port-au-Prince, Haiti; 2 Centers for Disease Control and Prevention, Health Reconstruction Team, Port-au-Prince, Haiti; 3 Center for Health Studies, Universidad del Valle de Guatemala, Guatemala City, Guatemala; 4 Centers for Disease Control and Prevention, Division of High-Consequence Pathogens and Pathology, Atlanta, GA, USA

**Keywords:** Dog bites, Haiti, rabies, socioeconomic

## Abstract

Haiti has the highest human rabies burden in the Western Hemisphere. There is no published literature describing the public's perceptions of rabies in Haiti, information that is critical to developing effective interventions and government policies. We conducted a knowledge, attitudes and practices survey of 550 community members and 116 health professionals in Pétionville, Haiti in 2013 to understand the perception of rabies in these populations. The majority of respondents (85%) knew that dogs were the primary reservoir for rabies, yet only 1% were aware that bats and mongooses could transmit rabies. Animal bites were recognized as a mechanism of rabies transmission by 77% of the population and 76% were aware that the disease could be prevented by vaccination. Of 172 persons reporting a bite, only 37% sought medical treatment. The annual bite incidence rate in respondents was 0·9%. Only 31% of bite victims reported that they started the rabies vaccination series. Only 38% of respondents reported that their dog had been vaccinated against rabies. The majority of medical professionals recognized that dogs were the main reservoir for rabies (98%), but only 28% reported bats and 14% reported mongooses as posing a risk for rabies infection. Bites were reported as a mechanism of rabies transmission by 73% of respondents; exposure to saliva was reported by 20%. Thirty-four percent of medical professionals reported they would wash a bite wound with soap and water and 2·8% specifically mentioned rabies vaccination as a component of post-bite treatment. The majority of healthcare professionals recommended some form of rabies assessment for biting animals; 68·9% recommended a 14-day observation period, 60·4% recommended a veterinary consultation, and 13·2% recommended checking the vaccination status of the animal. Fewer than 15% of healthcare professionals had ever received training on rabies prevention and 77% did not know where to go to procure rabies vaccine for bite victims. Both study populations had a high level of knowledge about the primary reservoir for rabies and the mode of transmission. However, there is a need to improve the level of knowledge regarding the importance of seeking medical care for dog bites and additional training on rabies prevention for healthcare professionals. Distribution channels for rabies vaccines should be evaluated, as the majority of healthcare providers did not know where rabies vaccines could be obtained. Canine rabies vaccination is the primary intervention for rabies control programmes, yet most owned dogs in this population were not vaccinated.

## INTRODUCTION

Rabies virus, a neurotropic lyssavirus which causes an invariably fatal encephalitis, affects more than 150 countries and territories [1]. Each year over 59 000 people die of rabies worldwide; which represents 160 deaths per day [2]. Rabies virus has differentiated into numerous variants, each associated with specific mammalian hosts. However, the viral variant associated with dogs is responsible for 99% of human rabies deaths worldwide. Many countries have recently made strides to eliminate the canine variant, and in 1983, the Pan American Health Organization (PAHO) launched a regional programme against rabies which has markedly reduced the incidence of human and canine rabies deaths in the Western Hemisphere [3]. Through the success of initiatives like those of PAHO, there are now only four countries in the Western Hemisphere that persistently record human deaths due to canine rabies virus: Haiti, Dominican Republic, Guatemala, and Bolivia.

In the Republic of Haiti, a Caribbean island nation of 10·5 million people, canine rabies virus variant remains a serious public health problem. An estimated 130 human deaths from rabies occur in the country each year [2]. Unfortunately, due to limitations in healthcare provider recognition, surveillance, and diagnostic capacity, only a fraction of these deaths are recognized [4]. The majority of recognized human rabies deaths and animal bite events have been reported from the West Department, which includes the capital city, Port-au-Prince. Almost all of these human deaths and bite events are due to domestic dogs; however, few successful preventive measures have been implemented due to lack of resources [4]. Haiti has an estimated 7 50 000 dogs according to a 2007 census, yet between 2009 and 2010, an average of only 2 14 627 dogs were vaccinated (annual vaccination rate of 28·6%).

A foundational component of a successful rabies programme is the formation of a well-educated population in regards to rabies risks and control of this disease [5]. Currently, no published studies have described the knowledge, attitudes and practices of rabies prevention in community members and healthcare professionals (HCPs) in Haiti. The Haitian Ministry of Health (MSPP) and US Centers for Disease Control and Prevention (CDC) conducted a community survey to better understand the risk of rabies exposure in Haiti, post-bite healthcare-seeking behaviours, and barriers to appropriate care.

## METHODS

A knowledge, attitudes, and practices survey was conducted in January–May 2013 (see Supplementary Appendix). Surveys were conducted in two populations within the commune of Pétionville: community members and HCPs. Pétionville had 359 956 inhabitants according to the National Institute of Statistics and Information (IHSI)’s 2012 estimates and is an urban community within the capital city Port-au-Prince. The Pétionville population has an above-average socioeconomic status [6]. Prior to conducting the survey, investigators held an orientation session for interviewers on the use of the questionnaire and consent form. Each questionnaire consisted of 47 (HCP) and 41 (community member) questions which were divided into five sections: Socio-demographic, Rabies knowledge, Attitudes towards animal bites, and Bite treatment practices. Surveys were conducted in Creole, Haiti's official language. Respondents were read questions, but were not provided answers from which to choose. Open-ended questions allowed for multiple responses.

### Community members

For the community survey a confidence level of 95% (1·96), a margin of error of 5% (0·05), a population-level knowledge of rabies estimated at 50% (because the proportion with knowledge of rabies was not known) (0·5) and design effect (1·5) were chosen to calculate the desired sample size: 576 participants. The sample size was increased by 20% to account for persons not eligible to participate due to age, absence from home or refusal to participate. A total sample size of 720 participants was determined necessary to detect statistically significant differences in responses.

A two-stage cluster sampling method was used utilized. Pétionville is administratively subdivided into 313 enumeration areas (EAs) by IHSI. Thirty (10%) EAs were randomly selected using the population proportionate-to-size method [7]. The quotient of 720 participants and 30 EAs was used to determine that 24 households were required from each cluster. In the 30 selected EAs, surveyors created an exhaustive list of all households using the continuous path of travel methodology to ensure that all households were included in the sample [7]. A random number generator was then used to randomly select 24 households from the list within the cluster. Each interviewer used a Global Positioning System unit to verify the boundaries of the cluster and to ensure that the correct houses were surveyed. Heads of households were requested for the interview; however, any willing participants aged ⩾18 years were eligible (one person interviewed per household).

### HCPs

For the survey of HCPs, we accessed a health facility registry for Pétionville, maintained by MSPP, to identify institutions eligible for the survey. Eligible health centres had either emergency outpatient, internal medicine or paediatric services. Forty-eight eligible facilities were identified. Health facilities were visited by interviewers on two consecutive days to recruit participants. Convenience sampling was conducted at all selected health facilities, with any doctor or nurse providing consent eligible for entry.

### Data collection and analysis

Two databases were created in Epi-Info v. 3.5.3 (CDC, USA) for data analysis with respect to the two study populations. Univariate analysis of frequency, rates, and means were calculated. Lifetime animal bite incidence was calculated as the number of primary bites that community participants reported receiving divided by the number of people surveyed. The annual bite incidence for the study population was determined by dividing the number of primary bites by the life-years represented in the study, which was calculated for each stratified age group. Respondents’ exact age was not collected, rather they were classified in an age group, and life-years were calculated as the product of the median age within each age group and the number of participants within the respective group.

### Ethical considerations

All study participants were aged ⩾18 years and signed a consent form. The study was approved by the Haiti Ethics Committee of the Haitian Ministry of Health (FWA 00016848) and the US CDC deemed the activity as non-research.

## RESULTS

### Community members

Of 720 individuals selected, 550 agreed to participate (response rate 76·3%). The average age of respondents was 35 (range 18–90) years (Table 1). There were more female respondents (56·5%) than males, and most respondents (71·5%) had at least secondary school education. Overall, 24% of respondents were unemployed, 22% were merchants, 13·8% were students, and the remaining reported other forms of employment. The annual median household income of the study population was 4274 USD, compared to a national median of 1800 USD. Pet ownership was documented for 229 respondents (41·6%), for which 453 pets were recorded. Dogs were more commonly owned (*n* = 239) than cats (*n* = 214). Of the 239 owned dogs, 91 (38·1%) had reportedly been vaccinated against rabies.

**Table 1.** Demographics of community members participating in a rabies knowledge, attitudes, and practices survey, Pétionville, Haiti, 2013

Community members (*N* = 550)			Healthcare professionals (*N* = 160)			Haiti National Statistics*
	*N*	%		*N*	%	
Median age, years (range)	35 (18–90)	−	Median age, years (range)	37 (23–72)	−	22·5 years
Median annual household income (range)	4274 USD (1200–14 400)	−		–	–	1800 USD
Sex						
Male	239	43·5	Male	15	14·2	50·1%
Female	311	56·5	Female	91	85·8	49·9%
Education						
Illiterate	50	9·1		–	–	22% of Haitians have a secondary degree or higher
Primary school	102	18·5		–	–	
Secondary school	318	57·8		–	–	
University	75	13·6		–	–	
No response	5	0·9		–	–	
Occupation						
Unemployed	132	24·0	Nurse	72	67·9	−
Professor	20	3·6	Doctor – social services	3	2·8	
Student	76	13·8	Doctor – general practice	18	17·0	
Merchant	123	22·4	Doctor – specialist	13	12·3	
Other employment	199	36·2		–	–	
Households owning pets						
Yes	229	41·6	Yes	51	48·1	-
Number of dogs	239	−	Number of dogs	51		
Rabies vaccinated	91	38·1†	Rabies vaccinated	13	25·5†	
Number cats	214	−	Number cats	0		
No	321	58·4	No	55	51·9	

* Source: *World Fact Book* [6].

† Percentage calculated as the quotient of rabies-vaccinated dogs and the total number of dogs owned by the survey respondents.

The majority, 85% (468), of respondents correctly reported that the primary rabies reservoir in Haiti was dogs (Table 2). Only 29·6% (163) reported cats as a vehicle of transmission, and only 1·5% were aware that bats or mongooses can also transmit rabies virus. Most respondents (75·6%) reported bites as a method of rabies virus transmission. One in five respondents reported that they were not aware of how rabies was transmitted. Regarding the prevention of disease, 71·6% (394) of respondents answered that rabies was a preventable disease through post-bite vaccination, and 79·5% (437) reported that vaccination of dogs and cats was an important strategy to reduce the transmission of rabies in humans. Nine out of ten respondents did not know where to go to receive rabies vaccination following an animal bite. Over half of respondents (54·7%) thought that rabies could be treated, even after symptom onset.

**Table 2.** Knowledge of rabies transmission and prevention of community members and healthcare professionals, Pétionville, Haiti, 2013

Rabies knowledge	Community members (*N* = 550)		Healthcare professionals (*N* = 106)	
	*n*	%	*n*	%
What animals can transmit rabies?*				
Dog	468	85·1	104	98·1
Cats	163	29·6	75	70·8
Other (bats, mongoose)	8	1·5	45	42·5
In what ways can rabies be transmitted to people?*				
Bite from an animal	416	75·6	78	73·6
Contact with saliva on open wounds	10	1·8	21	19·8
Consumption of raw meat from rabid animals	3	0·5	14	13·2
Don't know/No answer provided	110	20·0	5	4·7
Can post-bite vaccination prevent a person from developing rabies?				
Yes	394	71·6	91	85·8
No	113	20·5	15	14·2
Don't know	43	7·8	0	0
Do you know where to go to receive rabies vaccination if bitten by a suspected rabid animal?				
Yes	48	8·7	–	–
No	502	91·3	–	–
Can a person with rabies be cured after symptom onset?				
Yes	301	54·7	52	49·1
No	87	15·8	42	39·6
Don't know	162	29·5	12	11·3
Is vaccination of pets important for preventing human disease?				
Yes	437	79·5	104	98·1
No	51	9·3	2	1·9
Don't know	62	11·3	0	0
Can a person with rabies transmit the disease to other people?				
Yes	–	–	48	45·3
No	–	–	48	45·3
Don't know	–	–	10	9·4
Should anyone with a bite from a suspected rabid animal be considered for rabies vaccination?				
Yes	–	–	91	85·8
No	–	–	15	14·2
Does your institution have a protocol to guide in the treatment of bite victims?				
Yes	–	–	13	12·3
No	–	–	93	87·7
Do you know where to obtain rabies vaccine for a bite victim?				
Yes	–	–	24	22·6
No	–	–	82	77·4

* Multiple responses allowed, totals may not add up to 100%.

Overall 46% of respondents indicated that if they were bitten by a suspected rabid animal, they would kill the animal, and only 13% reported that they would take the offending animal to a veterinarian (Table 3). Very few respondents (8%) indicated that they would monitor the biting animal for signs of rabies. However, of the 172 persons (31·3% of total) who reported having had an animal bite in their lifetime, 80% said they did nothing with the offending animal, 16·3% said they killed the animal, and 8·7% said they observed the animal for 14 days. When asked what they would do if they were bitten by an animal suspected of having rabies, 90·7% said they would seek care at a clinic or hospital, 6% said they would wash the wound with soap and water, and 4·1% indicated they would do nothing or they did not know what they would do. No one reported that they would seek care from a traditional healer. Of the 172 bite victims captured in this survey, only 36·6% sought treatment at a clinic or hospital, and 8·1% washed the wound with soap and water. Over 36% of bite victims stated that they did nothing to treat the wound, and 3·5% of bite victims sought help from a traditional healer. Only 31·4% of bite victims said that they initiated the rabies vaccination series.

**Table 3.** Attitudes and practices towards bite events of community members, Pétionville, Haiti, 2013

Attitudes towards bites from suspected rabid animals (*N* = 550)			Practices of persons bitten by a suspected rabid animal (*N* = 172)		
	*n*	%		*n*	%
What would you do if bitten by a suspected rabid animal?*			What did you do after being bitten by the suspected rabid animal?*		
Nothing	3	0·5	Nothing	63	36·6
Wash the wound with soap and water	33	6·0	Washed the wound with soap and water	14	8·1
Seek help in a clinic or hospital	499	90·7	Sought help in a clinic or hospital	63	36·6
Self-administer antibiotics	3	0·5	Self-administered antibiotics	1	0·6
Visit a traditional healer	0	0·0	Visited a traditional healer	6	3·5
Don't know/Declined to answer	20	3·6	Don't know/Declined to answer	25	14·5
What would you do with an animal that had bitten you or a family member?*			What did you do with the animal that had bitten you?*		
Nothing	55	10·0	Nothing	138	80·2
Observe the animal for 14 days for signs of illness	44	8·0	Observed the animal for 14 days for signs of illness	15	8·7
Determine the rabies vaccination status of the animal	41	7·5	Determined the rabies vaccination status of the animal	2	1·2
Kill the animal	256	46·5	Killed the animal	28	16·3
Take the animal to a veterinarian for rabies assessment	73	13·3	Took the animal to a veterinarian for rabies assessment	3	1·7
Don't know/Declined to answer	81	14·7		–	–
			Did you receive rabies vaccination after the bite?		
			Yes	54	31·4
			No	110	64·0
			Don't know	8	4·7

* Multiple responses allowed, totals may not add up to 100%.

A total of 19 114 life-years are represented in this survey, of which 172 people indicated that they had experienced at least one animal bite (minimum annual bite incidence rate of 0·9%) (Table 4). The highest annual bite rate was seen in the 18–29 years age group (1·3 bites/100 life-years). The lowest bite rate was observed in persons aged ⩾60 years (0·13 bites/100 life-years). Dogs were the most frequently reported offending animal, responsible for 93·6% of bite events (Table 5). Of those interviewed, 16·9% indicated that they had been bitten by an animal inside their home, 49·4% by an animal at a neighbor's house and 33·7% reported experiencing a bite while on the street. The most common bite sites were lower extremities (84%), followed by upper limbs (11·6%).

**Table 4.** Annual and lifetime dog-bite incidence in community members, Pétionville, Haiti 2013

	Community respondents (*N*)	Life-years*	Primary bites reported (*N*)	Lifetime bite rate†	Annual bite incidence rate (bites/100 life-years)‡§
Age group, years					
18–29	266	6251	80	30·0%	1·28
30–39	121	4174·5	43	35·5%	1·03
40–49	66	2937	27	40·9%	0·92
50–59	50	2720	18	36·0%	0·66
⩾60	47	3031·5	4	8·5%	0·13
Sex					
Male	239	8365	81	33·9%	0·97
Female	311	10 885	91	29·3%	0·84
Total	550	19 114	172	31·3%	0·90

* Life-years were calculated as the product of the community respondents and the median of the age range.

† Lifetime bite rate was calculated as the quotient of the number of primary bites and the number of community respondents.

‡ Annual bite incidence rate was calculated as the quotient of the primary bites and the life-years represented by the study participants. The incidence rate is given as the number of bite events/100 life-years.

§ Because only primary bite events are represented in this table the lifetime bite rate and annual bite incidence rate represent a conservatively low estimate.

**Table 5.** Characteristics of persons bitten by suspected rabid animal, Pétionville, Haiti, 2013

	*n*	%
Survey respondents ever bitten by a suspected rabid animal	172 of 550	31·3
Average number of bites (range)	1·1 (1–3)	
What animal was responsible for the bite?*		
Dog	161	93·6
Cat	20	10·8
Age group (years)		
18–29	80	46·5
30–39	43	25·0
40–49	27	15·7
50–59	18	10·5
⩾60	4	2·3
Sex		
Men	81	47·1
Women	91	52·9
Where were you when bitten by the animal?*		
My home	29	16·9
Neighbour's home	85	49·4
On the street	58	33·7
On what part of your body were you bitten?*		
Lower limb	145	84·3
Upper limb	20	11·6
Chest	6	3·5
Face	2	1·2

* Multiple responses allowed, totals may not add up to 100%.

### HCPs

A total of 165 HCPs were approached for this survey, of which 106 consented (response rate 64·2%). The average age was 37 (range 23–72) years, and females represented 85·8% of respondents. Nurses comprised 67·9% of respondents and doctors 32·1% (Table 1).

Just over 98% of HCPs correctly identified dogs as the main transmitter of rabies virus in Haiti (Table 1). Cats were recognized as a risk for transmission by 70·8% of HCPs, and bats and mongooses by 42·5%. Bites were reported as a method of transmission by 73·6% of HCPs and direct contact the animal's saliva with mucous membranes or unhealed wounds was reported by 19·8%. Only 4·7% of HCPs were unaware of how rabies is transmitted.

Only 14·2% of HCPs reported having been trained regarding rabies prevention and bite management (Table 6). The majority (87·7%) of HCPs reported that their institutions did not have a protocol for the treatment of bite victims or rabies vaccination and 77·4% were not aware of where to obtain rabies vaccine for bite victims (Table 2). When asked if people bitten by a suspected rabid animal should receive post-exposure prophylaxis, 85·8% responded ‘yes’. However, when asked what care they would recommend for a person with a suspected rabies exposure nearly 40% (39·6%) of HCPs responded that they did not know. Wound washing was mentioned as a post-bite treatment option by 36% of HCPs and tetanus toxoid injection by 17·9%. Rabies vaccination was only mentioned as a post-bite action by 2·8% of HCPs (Tables 2 and 6).

**Table 6.** Practices of healthcare professionals when treating bite wound victims, Pétionville, Haiti, April 2013

Survey questions	*n*	%
Have you ever received training on rabies control and prevention?		
Yes	15	14·2
No/Unknown	91	85·8
Do you need to alert local authorities when you treat a bite victim?		
Yes	101	95·3
No/Unknown	5	4·7
Do you discuss rabies with patients seeking treatment for an animal bite?		
Never	39	36·8
Sometimes	53	50·0
Often	9	8·5
Almost always	5	4·7
What treatment do you recommend to patients bitten by an animal suspected of having rabies?*		
None	0	0
Immediately wash the wound with soap and water	36	34·0
Administer a tetanus vaccine	19	17·9
Administer antibiotic treatment	6	5·7
Provide rabies post-exposure prophylaxis	3	2·8
Don't know/Declined to answer	42	39·6
What do you recommend to patients bitten by unfamiliar animals when your institution does not have rabies vaccine in supply?*		
Nothing	1	0·9
Tell the patient to return home	1	0·9
Refer the patient to another centre where vaccine is available	63	59·4
Call another institution to see if the vaccine is available	11	10·4
Alert the health authorities in the region	13	12·3
Don't know	21	19·8
What do you recommend bite victims do with the animal which has bitten them?*		
Observe the animal for at least 14 days	73	68·9
Check if the animal has been vaccinated against rabies	14	13·2
Kill the biting animal	2	1·9
Take the animal to the veterinarian for a health assessment	64	60·4
Don't know	7	6·6
When should a person be given rabies vaccine?*		
If the animal was confirmed to have rabies	34	32·1
If the biting animal cannot be located or was unfamiliar to the bite victim	19	17·9
If the biting animal died or was killed shortly after the bite	21	19·8
If saliva made contact with broken skin or a mucous membrane (i.e. a bite)	15	14·2
Don't know	39	36·8

* Multiple responses allowed, totals may not add up to 100%.

## DISCUSSION

This is the first assessment of rabies knowledge, attitudes, and practices in community members and medical professionals in Haiti. The study was conducted with three objectives: (i) to improve understanding of rabies exposure risks in Haiti, (ii) describe post-bite healthcare-seeking behaviours, and (iii) describe barriers to appropriate treatment. The timing of this study is unique, in that it describes these risks and behaviours prior to the development of national bite treatment and rabies vaccination guidelines. Based on the results of this study, recommendations were drafted in 2014 and HCP training began in early 2016. Findings presented here address the study objectives as well as serve as a baseline for future studies by which the impact of the national guidelines can be measured.

### Rabies exposure risk

Rabies is a zoonotic disease, and in Haiti it is primarily spread through the bite of an infected dog [8]. Therefore, two conditions must be satisfied for a rabies exposure to occur: a rabid dog and a bite event. The probabilities of these two events occurring is the basis for many probabilistic rabies burden models [2]. A 2015 surveillance publication reported that the rate of rabies in biting dogs was 7%; however, at the time of this report the community bite rate was not known [9]. This survey reports an annual bite rate of 0·9% (a comparable figure to what has been reported in other developing countries) [10–13]. The annual bite rate was highest in the 18–29 years age group and declined with each following age group (Table 4). Bite incidence data for persons aged <18 years was not collected; however, the trend observed here is consistent with WHO published literature which equates a higher bite risk in younger age groups [14]. One in three bites occurred on the street; these exposures likely represent a higher rabies risk due to higher rates of rabies transmission in free-roaming dogs [9, 15].

The bite rate reported here is over three times lower than a bite rate reported by Schildecker *et al*. in 2016 in which dog owners attending a Haitian vaccination clinic were assessed (3·1% bite rate for dog owners) [16]. There are several likely explanations for the lower bite rate in the present study. First, this is a general community survey, whereas the previous study [16] focused only on dog owners, who may have more frequent contact with dogs and therefore increased risk for a bite event. Second, this survey asked for all bite events in the respondent's lifetime, which is prone to recall bias and may underestimate the true bite rate. Last, the bite rate is likely to be higher in children aged <18 years, which was not addressed in this study. The findings reported here, when considering Schildecker *et al*.’s findings, support the view that the dog bite rate in Haiti is not static and likely dependent upon dog ownership and the community in which an individual resides. The bite rate reported here is likely an underestimate, but potentially less biased than the population assessed by Schildecker *et al*. [16]. Therefore, utilizing the generalized community bite rate provided by this study is probably most appropriate for probabilistic modelling of rabies burden. However, more complex burden efforts should take into consideration that the risk for bites may be higher in certain populations.

### Healthcare-seeking behaviours

Rabies is a preventable disease when appropriate post-bite care is provided. Therefore, even in the face of a high risk for rabies exposure in Haiti, disease can be mitigated through appropriate healthcare-seeking behaviour and wound treatment. Unfortunately, findings from this study paint a bleak picture of healthcare-seeking behaviour in dog-bite victims. While nine out of ten individuals surveyed reported that they would seek medical care if they were bitten by an animal, in practice, fewer than four in ten people sought medical care after being bitten. Pétionville is one of the wealthiest communities within Haiti and, as expected, our study population had a household income over three times the national average [6]. Presumably, higher income families and communities have increased access to healthcare. Therefore, post-bite healthcare-seeking behaviours are likely to be even lower in many parts of Haiti, and figures presented here represent a ‘best-case scenario’.

Knowledge of rabies did not appear to be the primary reason for the observed low healthcare-seeking behaviours. Most respondents (85·1%) knew that rabies is transmitted by dogs, via a bite (75·1%), and that rabies is preventable by vaccination (71·6%). But there were several educational deficits noted; few people recognized that washing the wound with soap and water can reduce the risk of rabies and only 15·8% of persons knew that rabies is fatal after symptom onset. These represent potential areas to engage communities through educational campaigns. However, healthcare outreach can be particularly difficult in developing world settings, where literacy and education levels are frequently encountered barriers [10, 17, 18]. In Haiti only 61% of the population is literate, compared to 92% in other Latin American countries, and only 10% of Haitians have access to the internet [6, 19]. Educational campaigns that utilize printed materials in combination with verbal engagement (i.e. radio) in lieu of more technologically advanced communication mediums may be more successful in reaching community members in Haiti. Integration of rabies prevention and control messaging into school curriculums may also be a successful strategy, as literacy rates may be higher in primary- and secondary-school children.

While healthcare-seeking behaviours were low, data suggests that when a person sought medical care they were very likely to receive rabies vaccination; 54 of 63 bite victims that sought medical care self-reported that they received rabies vaccination. However, other data from this study conflict with this self-reported vaccination rate. For example, only 23% of HCPs even knew where to obtain rabies vaccine and only 2·8% listed rabies vaccination as a standard component of post-bite care. Survey methodology utilized open-ended questions which can prevent the respondent from falsely identifying information that is revealed by the interviewer. Unfortunately, this design limits our ability to state that HCPs would not have provided rabies vaccine to the hypothetical bite victim. When asked directly if rabies vaccine should be provided for persons bitten by a suspected rabid dog, nine of ten HCPs answered affirmatively. In this respect, the HCP inconsistency regarding rabies vaccination is likely due to survey design.

Given the apparent difficulty in locating rabies vaccine in Haiti and questionable provider response rate for administering the vaccine, it is difficult to imagine that rabies vaccine administration is as high as the self-reported rate identified here. It may be likely that persons who sought medical care were unaware of what they had received; a probable scenario, as tetanus and antibiotics were much more frequently reported by HCPs as standards of bite treatment compared to rabies vaccine. Anecdotally, it is common for bite victims in Haiti to be unaware of what they were treated with; often confusing a one-time tetanus shot with the rabies vaccine. Furthermore, this study did not assess how many doses nor the dosing schedule that the bite victims adhered to, both are critical to prevent development of rabies. A more robust method for describing rabies vaccination practices and adherence is warranted, such as a prospective healthcare-based study and a systematic evaluation of the rabies vaccine distribution system.

Perhaps the most pronounced barrier to rabies prevention identified in this study pertained to HCP training; fewer than 15% of HCPs reported that they had received training regarding rabies prevention. At the time of this survey in 2013 Haiti had no national recommendations for the treatment of bite wounds nor recommendations for the prescription of rabies vaccination. Probably a reflection of this lack of recommendations and training, 50% of HCPs thought that rabies was treatable after symptom onset and 40% stated that they did not know what care they should provide to a bite victim. In 2014 preliminary findings from this study were shared with MSPP, and in response, PAHO assisted in the development of a healthcare provider education programme on rabies. As of 2016, this training programme had been implemented in selected areas, but had not yet been nationally disseminated. The impact of these guidelines should be measured in the future and compared to the findings from this study.

Haiti has a fragmented healthcare delivery system, and this impacts the distribution and availability of rabies vaccine. Human rabies vaccine is typically donated by international governments to MSPP, which distributes the vaccine to only 16 health centres, out of more than 116 sentinel hospitals [4]. These 16 health centres often report being out of stock of the vaccine, and national shortages have occurred over the past 5 years. Vaccine can be procured from some private pharmacies at a cost of 25 USD per dose, the equivalent of 2 months wages for a complete five-dose course for the average Haitian family. Even with improvements in bite treatment training, shortages of vaccines and deficiencies in vaccine distribution may still prohibit appropriate vaccination of bite victims. Therefore, evaluation of existing vaccine distribution channels and enhancing rabies vaccine distribution should be considered.

### Rabies prevention and animal vaccination

Many survey respondents recognized the importance of canine rabies vaccination in disease control. However, in practice, only 35·9% of community respondents’ dogs were vaccinated against rabies (an even lower vaccination rate was observed in dogs owned by HCPs). This represents a gap in knowledge and practice of animal rabies vaccination, and several factors may be responsible. Haiti has very limited veterinary capacity with fewer than 50 veterinarians for the entire country's 10·5 million people; therefore, access to routine animal care, including rabies vaccination, is unlikely for many pet owners. After the 2010 earthquake, access to medical supplies, including rabies vaccines for animals, declined sharply. Currently, for most Haitians, animal rabies vaccine is only available during government mass vaccination campaigns. When these campaigns are not held, animal rabies vaccine may be almost impossible to procure. Furthermore, the average Haitian survives on less than 3 USD per day; therefore, despite awareness of the benefits of rabies vaccination, payment for veterinary services may not be feasible [6]. The average income of our study population was three times the Haitian national average, so cost of vaccine may not have driven the low vaccination rates in this community, rather access to the vaccines may be an important factor. Increasing the frequency and accessibility of dog rabies vaccination programmes, in a manner which considers the high poverty levels in Haiti, is probably needed to see increases in dog vaccination coverage.

This study has several limitations. First, it was focused in one commune within the capital city, Port-au-Prince, and therefore may not be representative of all Haitian communities. Pétionville is one of the wealthier communes in Haiti, therefore access to education and health services may be better compared to the majority of Haitians. In that respect, the results from this survey may represent a ‘best-case scenario’. In addition, the survey of HCPs was a convenience sample, and therefore the HCPs interviewed may not be representative of all HCPs in Pétionville.

In conclusion, this study identified that in community members and HCPs in a community at a relatively high socioeconomic level for Haiti, large disconnects exist between rabies knowledge and practices. As a promising sign, after results of this survey were reported to MSPP, national bite treatment guidelines were developed and a healthcare provider education programme was implemented in select communities. These training programmes for HCPs should be evaluated and, if effective, expanded. Difficulties in access to both human and animal rabies vaccination was evident for this study population, and efforts should be made to make these vaccines more accessible within the country. With the high rate of rabies exposure in Haiti, an educated healthcare workforce and population will be necessary to ensure appropriate treatment for persons exposed to rabid animals.

## Supplementary material

For supplementary material accompanying this paper visit https://doi.org/10.1017/S0950268816003125. click here to view supplementary material
