# Supplementary material for: Knowledge, attitudes and practices regarding rabies risk in community members and healthcare professionals: Pétionville, Haiti, 2013
Source: Epidemiol Infect. 2017 Mar 14;145(8):1624–34. doi: 10.1017/S0950268816003125 (PMC5426290; doi:10.1017/S0950268816003125)
Supplement: Supplementary file 1 [file S0950268816003125sup001.docx]

**APPENDIX**

**Knowledge, Attitudes and Practices of the population on rabies risk**

Hello / Good evening, my name is ______, I work for the Ministry of Public Health and Population (MSPP) as part of the training program in epidemiology of field (FETP). The Ministry is carrying out a study for information on knowledge, attitudes and practices of the population on the risk of transmission of rabies in the town of Pétion Ville. This study will help us acquire information on the attitudes of the population after having been bitten by an animal suspected of rabies and their behavior towards the management. The results of this study will enable the Ministry to identify measures of prevention and control to reduce the transmission of rabies at the community level. The answers you give will be confidential and used only for this study. This questionnaire counts _____ questions, we ask you for about 30 minutes to answer the questions. Your participation is entirely voluntary and your name will not be noted on the questionnaire. Thank you very much for your participation.

Do you accept to participate in the study? **Yes-1 no-0**

**Interview control sheet, completed by the interviewer**

(1) Question Number: | _ | _: _ | _ | _ |

(2) Interviewer’s Number:: _: _ |

(3) Date of interview: ____ / _ / 2012 (dd/mm/yyyy)

(4) Interviewer : ___________________________________________

(5) BDS number: ___ / ___ / ___

(6) Municipal section: _

(7) Participant’s consent Yes-1 No-0

**Instructions:**

•         Please carefully read each question for the participant

•         Circle the matching number has the answer given by the participant

•         Don't read the answers to the participants.

**Remember that your answers will be confidential.**

**SECTION 1: Now we will discuss on general or demographic information.**

| **N^o^** | Questions | Answers |
| --- | --- | --- |
| **Q8** | What is your age?  If the respondent is under 18, ask if there is another adult in the house. | Birth year _____ / _____ years |
| **Q9** | What is your sex? | Male 1 Female 2 |
| **Q10** | What is your level of education? | Has never been a school 0 Elementary 1 Primary 2 Secondary 3 University 4 Won't answer 99 |
| **Q11** | What is your occupation? | None 0 Teacher 1 Student 2 Trader 3 Driver 4 Maid 5 Home worker 6 Other (specify) ___88 Won't answer 99 |
| **Q12** | How many people live in this household? | ___ |
| **Q13** | How many people are employed in this House? | ___ Won't answer 99 |
| **Q14** | What is your monthly household income? | Less than 4800 g 1 4801 - 9999 g 2 10000 - 39999 gourdes 3 More than 40000 4 Won't answer 99 |
| **Q15** | With what material is your home built with? | Blocks/stones, ceramic and 1 concrete roof Blocks/stones, cement and  2 concrete roof Blocks, cement, and plate 3  Other (specify) ___88 |
| **Q16** | Do you have pets in the House? | Yes 1 No 0 |
| **Q16a** | If Yes, what kind? | Dog 1 Cat 2 Goats 3 Pig 4 Other (specify) ___88 |
| **Q16b** | How many: | Dogs _ Cats _ |
| **Q17** | Have all animals have been vaccinated against rabies? | Yes 1 No 0 |
| **Q18** | If Yes, when were they vaccinated? | _____ month _____ year |

**General comments:
Cars belonging to the household___, Dimension of the house___, physical condition of the house___**

**SECTION 2: Now, we'll ask you some questions about your knowledge of rabies.**

| **Q19** | What are the animals according to you who might give rabies? | Dog 1 Pig 2 Cat 3 Horse 4 Bat 5 Mongoose 6 Other (specify) ___88 Don't know 98 |
| --- | --- | --- |
| **Q20** | According to you, how is rabies transmitted? | Bite or scratch by an animal suspected of rabies. 1 Direct contact with the saliva of an animal with an unhealed wound on the skin. 2  By consuming raw meat of animals suspected of rabies. 3 Direct contact with the saliva of  an animal with intact skin 4 Other (specify) ___88  Don't know 98 Won't answer 99 |
| **Q21** | Do you believe that rabies can be prevented? | Yes 1 No 0 Don't know 98 |
| **Q22** | Do you believe that vaccination of animals (dogs, cats) could prevent rabies in humans? | Yes 1 No 0 Don't know 98 |
| **Q23** | Do you think that rabies is a curable disease? | Yes 1 No 0 Don't know 98 |
| **Q24** | In your opinion, the administration of adequate prophylactic treatment (vaccine and rabies serum) can prevent a person bitten by an animal suspected of rabies from developing rabies? | Yes 1 No 0 |
| **Q25** | In your opinion, do you think the elimination of stray dogs can reduce the transmission of rabies in the communities | Yes 1 No 0 |
| **Q26** | Do you know in which institution the rabies vaccine is available in your area | Yes 1  No 0 |
| **Q26a** | If answered Yes, which institutions | _________________________ |

**SECTION 3: Now, we'll ask you some questions about your attitudes of rabies.**

| **Q27** | If you or your child is bitten by an animal suspected of rabies, What will you do? | Nothing 0 Wash immediately with water and soap 1 Look for help at a clinic or hospital 2 Visit to a private doctor 3  Administer antibiotics yourself 4 Visit a public doctor 5 Ask for an anti-tetanus vaccine 6  Other (specify) ___88 Don't know 98 won't answer 99 |
| --- | --- | --- |
| **Q28** | In your opinion, if you or your child is bitten by an animal suspected of rabies. What will you do with this animal | Observe the animal for two weeks at least 1 Check if the animal has been vaccinated against rabies 2  Kill the animal 3 Take the animal to the vet to check if it is infected 4 Other (specify) ___88 Don't know 98 Won't answer 99 |
| **Q29** | If in your community, you suspect someone has been bitten by an animal suspected of rabies. Do you think it's necessary to alert local health authorities? | Yes 1 No 0 Don't know 98 |
| **Q30** | Do you usually talk or discuss with your family or neighbors about the prevention of rabies? | Never 0 sometimes 1 Often 2  Very often 3 |

**SECTION 4: The following questions are intended to describe your practices about rabies**

| **Q31** | Have you been bitten by an animal at least once? | Yes 1 No 0 |
| --- | --- | --- |
| **Q32** | If Yes, what kind of animal? **If no, proceed to the question** | ______________________ |
| **Q33** | How many times | ____/____ |
| **Q34** | What was the last time that you have been bitten by a dog or cat? | < 1 month From 1 to 6 months From 6 months to 12 months > 12 months Don't know 98 |
| **Q35** | Where were you bitten (place)? | Doesn't remember 0 Home 1 The neighbor 2 In the Street 3 Other (specify) 88 |
| **Q36** | In what part of the body have you been bitten? | Face 1 Upper limb 2 Lower limb 3 Chest 4 Other (specify) ___88 |
| **Q37** | Once you've been bitten by this animal. What did you do? | Nothing 0 Wash immediately with water and soap 1 Search for help at a clinic or hospital 2 Visit to a private doctor 3  Administer antibiotics yourself 4  Visit a doctor sheet 5  Ask for an anti-tetanus vaccine 6  Other (specify) ___88  won't answer 99 |
| **Q38** | Once you've been bitten, What did you do with the biting animal? | Nothing 0 The animal was put under observation for two weeks 1 Verified that the animal was vaccinated 2 The dog was sacrificed (killed) 3 We took the animal to the vet to check if he was infected 4  Other (specify) 88 Won't answer 99 |
| **Q39** | Have you received a preventive rabies treatment? | Yes 1 No 0 Don't know 98 |
| **Q40** | Have you received all doses of the treatment? | Yes 1 No 0 |
| **Q41** | If no, what is the reason you have not completed all doses of treatment? | Nothing 0 Abandonment 1 Out of stock at the hospital 2 It was not necessary to take all the doses 3 Other (specify) ___88 |
| **Q42** | If you have been bitten by a suspected animal of rabies, and the rabies vaccine was not available and you had been referred to another institution, what did you do? | Return home without anything 1 Go to the institution where the vaccine is available 2 Other (specify) ___88 Won't answer 99 |
| **Q43** | Have one of your children or loved ones was bitten by an animal suspected of rabies at least once? | Yes 1 No 0  Don't know 98 |
| **Q44** | If Yes, what do you do? | Nothing 0 I washed the wound with water and soap1 I drove him to the hospital  I took him to a doctor sheet 3 I asked a tetanus vaccine for him 4 Other (specify) ___88 |

**SECTION 5: Questions on program interventions against rabies**

| **Q45** | During the last 30 days, how many messages on prevention of rabies have you seen or heard on the radio, television, newspapers or signs? | None 0 Little 1 A lot 2 Won't answer 99 |
| --- | --- | --- |
| **Q46** | During the last 30 days, how did you hear about rabies? | None 0 Radio 1 TV 2 Newspapers 3 Signs 4 Zone health worker 5 |
| **Q47** | In your opinion, what are the best ways to receive new information on the prevention of rabies for you and your family? | Broadcast messages 1 Billboards 2 TV 3 Posters 4  Flyers 5 Through health workers in zone 6 |
